# Supplementary figures and images for: Mortality after emergency unit fluid bolus in febrile Ugandan children
Source: PLoS One. 2023 Aug 31;18(8):e0290790. doi: 10.1371/journal.pone.0290790 (PMC10470955; doi:10.1371/journal.pone.0290790)

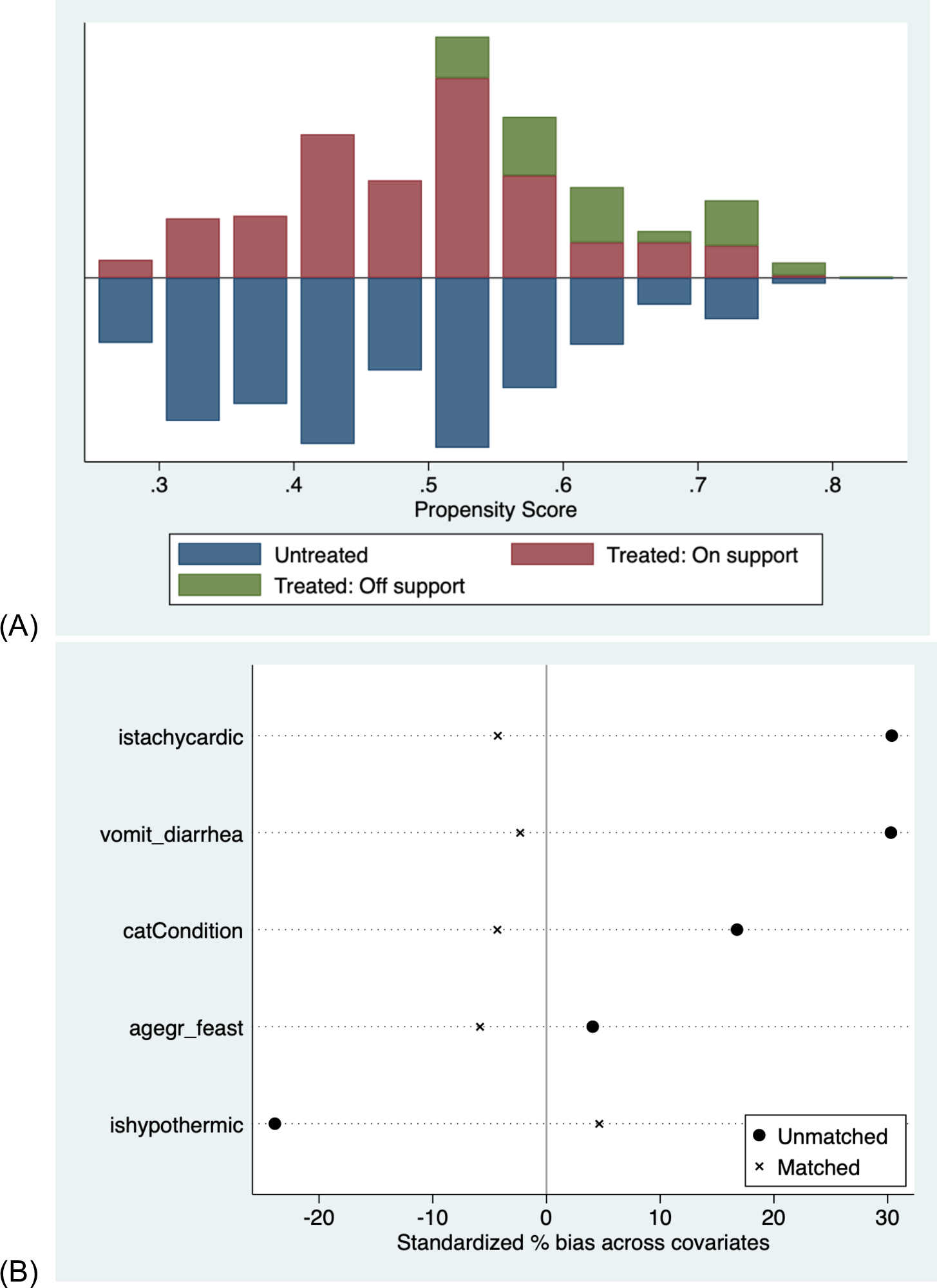

Supplement: S1 Fig — (A) Graphical representation of patients that are on and off support for propensity score matching using psmatch2 in Stata 16. (B) Graphical representation of standardized bias in the variables included in propensity score matching before and after matching using psmatch2 in Stata 16. (TIF) [file pone.0290790.s001.tif]
