# Supplementary material for: Mortality after emergency unit fluid bolus in febrile Ugandan children
Source: PLoS One. 2023 Aug 31;18(8):e0290790. doi: 10.1371/journal.pone.0290790 (PMC10470955; doi:10.1371/journal.pone.0290790)
Supplement: Attachment — Submitted filename: Reviewer Comments.docx [file pone.0290790.s002.docx]

Dear Reviewers and Editors,

We thank you for the opportunity to revise and resubmit our manuscript “Mortality after emergency unit fluid bolus in febrile Ugandan children”. We appreciate your close reading and excellent feedback and have addressed your comments below in a point-by-point manner.

Thank you again for your time and consideration.

Sincerely,

Brian Rice

General

1. PLOS ONE’s style requirements have been met for formatting.
2. A complete copy of PLOS’ questionnaire on inclusivity in global research has been attached.
3. The ethics statement in the Methods has been changed to “Ethics review board approval and waiver of patient consent was provided by the Mbarara University of Science and Technology Institutional Review Committee (No. 11/08-12) and University of Massachusetts Institutional Review Board (#14570).”
4. The same paragraph as #3 was added to expand our Ethics methods “Ethics review board approval and waiver of patient consent was provided by the Mbarara University of Science and Technology Institutional Review Committee (No. 11/08-12) and University of Massachusetts Institutional Review Board (#14570).”

| Reviewer #1 | |
| --- | --- |
| Major | |
| 1. The specific inclusion and exclusion criteria should be state din the methods (the exclusion of patients by not being admitted is currently in the results) | They have been moved to Methods, and Results changed to “Of those, 3,087 patients met inclusion and exclusion criteria listed in Methods above.” |
| 2. It seems strange to say that in MVR, fluids were not associated with increased or decreased mortality. This use of increased and decreased occurs in multiple places throughout the results and discussion, and it feels a bit redundant. Perhaps it would make more sense to say there was no association with mortality. I acknowledge that this doesn’t provide directionality, but I think it still conveys the message. | The statement “increased or decreased” has now been removed in all cases except the first time the result is described in “In all four logistic regression models, fluids were *not found to be significantly associated with increased or decreased mortality”* |
| 3. The authors suggest that looking at mortality further out (for example 28-day) may be helpful. Is there a study doing this, particularly for those admitted, and was there ongoing additional resuscitation. These are just general thoughts, but if this is being investigated, it would be worthwhile to state this. | We share the reviewer’s interest in longer term outcomes, but the QA database this data comes from was originally built to look only at short term outcomes and doesn’t contain 7 or 28-day outcomes. |
| Minor: - There are several grammatical errors throughout. - Line 251 – “There no…” it seems there is a word missing. - On line 316 “our manuscript provides…..” This should be “our study provides” | Line 251: changed to “There was no…”  Line 316: “manuscript” has been changed to “study” |
| Reviewer #2 | |
| Major | |
| Suggest including absolute risk difference in the abstract: The authors should consider including the absolute risk difference with 95% confidence interval in the abstract (from at least one of the analyses), rather than simply stating “No significant mortality benefit or harm.” The upper and lower limit of the 95%CI of the mortality difference would help the reader understand the precision of the null finding (and judge whether a larger study is needed). | The abstract has been edited to include this information. |
| Comment (please address with a sentence or two in Discussion or Limitations) Lacking are details of the fluid resuscitation, which may be important for explaining differences with FEAST. “Fluids” versus “no fluids” as a binary variable is a bit simplistic, given that there is a range of appropriate and inappropriate IV fluid therapies. The patient weight was only recorded in ~50%, so the mL/kg fluid dose could not be calculated for a large fraction of patients. The mean dose of fluids was 25.5 mL/kg (standard deviation 19.9), meaning that some patients received much more than 20mL/kg (recommended “bolus” fluid volume). The authors appropriately refrained from more in depth descriptive analyses of the fluid administration because of the limited quality of the data; however, these details may be important. The type of fluid was not specified (I could not find this in the paper) – normal saline? Ringer’s lactate? The frequency of IV fluid administration (or continuous infusion) was not specified. In a high-income setting, “maintenance” fluids are often administered after an initial bolus, in addition to fluids to correct the deficit at presentation. I acknowledge that, in the LMIC context, careful reassessment of fluid requirements may exceed nursing or medical capacity, but a single episode of fluid administration does not seem physiologic (frequent reassessment needed, these are critically ill patients). Was the IV fluid given once and never again? For critically ill patients, ongoing IV fluid input may be required before enteral feeds can be started. At what rate was the fluid given (“bolus” push, mL/hour)? This could be important if endothelial leak, pulmonary edema, and/or cerebral edema contribute to increased mortality with rapid fluid administration. To address this limitation, I suggest that the authors add a sentence or two to the limitations paragraph, highlighting the details of the fluid therapy that would be helpful (e.g., in future studies) to better define the “exposure.” | Additional details were added into the results “Of patients that got fluids, 1050 received normal saline, 462 received lactated Ringer’s solution, and 12 received a mix of both. Looking at the patients that got fluids and had a recorded weight, 22.1% received less than 20 cc/kg (n=170), 60.6% (n=466) received between 20-29 cc/kg, and 17.3% (n=133) received 30 or more cc/kg. The mortality within each of those three groups was 4.7% (n=8), 3.9% (n=18) and 5.3% (n=7) and was not statistically significantly different (p=0.7).”  We also added to the Limitations section “Furthermore, because rate of administration was not recorded in this data the analysis was unable to address the contribution of rapid (bolus) versus continuous (maintenance) fluid administration to mortality.” |
| Comment (no change needed) Overall, the quality of the clinical data is impressive (presence of missing data was low, ≤ 3.0% across all variables). However, given that weight was only recorded in 50% (this is an important clinical parameter in pediatric medicine for dosing of all kinds of medications, not only fluids), one cannot help but harbour lingering doubts about the accuracy and thoroughness of ?paper records collected in a ED in a rural hospital in an LMIC for clinical purposes. Perhaps additional detail on the added human resources (“trained research assistants”) provided to the hospital over the years would be helpful to assuage some misgivings about the quality of retrospective clinical data. These data just seem surprisingly “clean” compared to other Ugandan hospital records I have worked with. The staff should be congratulated on such meticulous record keeping. | We agree that the fidelity of the data is remarkable but would like to assure the reviewer that it is very genuine and something which a huge amount of effort and human resources were applied to over a decade. To help explain how this was accomplished we added the following to Methods “The six-bed emergency unit was staffed by at least one (and typically two) clinicians and at least one nurse and one nursing student during the entire operating hours from 0800 – 2400. Additionally, a trained research assistant was present in the emergency unit during all operating hours who input paper charts into the electronic database while the patient was still in the emergency unit. This staffing situation allowed for a very high fidelity of data capture. Digital scans were then made of every chart to allow review as needed.” I can also assure the reviewer that we had an additional layer of data quality as we scanned all the paper charts over ten years and had them for remote review along with in-country review of the hard copies. The author team has done multiple rounds of hand reviewing and spot checking with those scanned copies over the years and the data is actually of the reported quality. |
| Using a “gestalt” of how sick the patient was, versus a standardized composite clinical severity score: Clinical impression (“not sick,” “sick,” or “toxic”) was a highly prognostic variable. I agree that this assessment, when performed by a trained or experienced clinician, is valid. The authors should also consider using one of several published, standardized, validated composite severity scores such as LODS (works for non-malarial febrile illness – see for example Conroy AL, et al. Prospective validation of pediatric disease severity scores to predict mortality in Ugandan children presenting with malaria and non-malaria febrile illness. Crit Care. 2015; 19(1): 47), SICK (Signs of Inflammation in Children that Kill), or PEDIA. There are even composite severity scores derived from the FEAST cohort (FEAST Paediatric Emergency Triage (PET) Score). The purpose of using one of these would be to have an objective and standardized scale of how sick the patient was. Important to adjust effect of fluids on mortality for disease severity at baseline, which the score would do nicely. This is a discretionary revision. | We would like to thank the reviewer for this suggestion. Looking at how non-clinician gestalt performs is something that has not been done for emergency medicine to our knowledge and is a very intriguing concept. Given that the current manuscript is already relatively methods heavy with several subgroup and sensitivity analyses we will look at a separate manuscript to pursue gestalt analysis in a robust manner. |
| Comment (positive, no change needed): The analysis (propensity score matching, multiple imputation for missing data, subgroup analysis of patients meeting inclusion criteria for FEAST) appears to be masterfully executed. This thorough analysis was indeed necessary, despite the “null” result, since these findings invite many questions in the shadow of FEAST. I appreciated the transparent and critical analysis of the sample size (post hoc power). This clearly and completely addressed the question that arose as I read the manuscript about the power of a study with “null” conclusion (risk of type 2 statistical error). The power calculation indicated that the study was underpowered with respect to the relative increase in mortality seen in FEAST (but adequate to rule out the absolute mortality increase seen in FEAST), despite the large number of patients included over an 8 year period (though with only 57+66 events, 4% mortality). | Thank you so much for your kind comments. |
| Comment (no change needed) The authors attempt to explain difference in mortality at the rural hospital (4%) compared to FEAST centres (9.5%): “Other possibilities include different care seeking behavior for this rural emergency unit than in the urban hospital inpatient settings used to enroll patients in FEAST.” I agree that, for example, Mulago Hospital in Kampala (a FEAST centre) is a national referral hospital and receives the sickest patients from across the country, including those that could not be managed at peripheral centres like Karoli Lwanga Hospital and many others. The patients are a select group of the sickest, that have previously failed standard management. The higher mortality in FEAST is not surprising to me. | Thank you for your insight and understanding of the heterogeneity of care settings within Uganda. |
